# Membrane Disintegration Caused by the Steroid Saponin Digitonin Is Related to the Presence of Cholesterol

**DOI:** 10.3390/molecules201119682

**Published:** 2015-11-09

**Authors:** Ikhwan Resmala Sudji, Yamunadevi Subburaj, Nataliya Frenkel, Ana J. García-Sáez, Michael Wink

**Affiliations:** 1 Department of Biology, Institute of Pharmacy and Molecular Biotechnology, Heidelberg University, INF 364, 69120 Heidelberg, Germany; ir.sudji@gmail.com; 2 Interfaculty Institute for Biochemistry, University of Tübingen, 72076 Tübingen, Germany; yamunabio@gmail.com (Y.S.); ana.garcia@uni-tuebingen.de (A.J.G.-S.); 3 Max Planck Institute for Intelligent Systems, 70569 Stuttgart, Germany; 4 Physical Chemistry of Biosystems, Institute of Physical Chemistry, Heidelberg University, 69120 Heidelberg, Germany; n.frenkel@stud.uni-heidelberg.de; 5 Institute for Toxicology and Genetics, Karlsruhe Institute for Technology, 76021 Karlsruhe, Germany

**Keywords:** digitonin, cholesterol, calcein release, giant unilamellar vesicles, dynamic light scattering, membrane permeability

## Abstract

In the present investigation we studied the molecular mechanisms of the monodesmosidic saponin digitonin on natural and artificial membranes. We measured the hemolytic activity of digitonin on red blood cells (RBCs). Also different lipid membrane models (large unilamellar vesicles, LUVs, and giant unilamellar vesicles, GUVs) in the presence and absence of cholesterol were employed. The stability and permeability of the different vesicle systems were studied by using calcein release assay, GUVs membrane permeability assay using confocal microscopy (CM) and fluorescence correlation spectroscopy (FCS) and vesicle size measurement by dynamic light scattering (DLS). The results support the essential role of cholesterol in explaining how digitonin can disintegrate biological and artificial membranes. Digitonin induces membrane permeability or causes membrane rupturing only in the presence of cholesterol in an all-or-none mechanism. This effect depends on the concentrations of both digitonin and cholesterol. At low concentrations, digitonin induces membrane permeability while keeping the membrane intact. When digitonin is combined with other drugs, a synergistic potentiation can be observed because it facilitates their uptake.

## 1. Introduction

Saponins are natural glycosides, which are widely distributed in the plant kingdom and in some marine animals like sea cucumbers (Holothuriidae) and sea stars (Asteroidea) [1,2]. Saponins are mainly divided into monodesmosides with a single sugar chain and bidesmosides with two sugar chains. Monodesmosides exhibit strong membrane activities which are due to their amphiphilic molecular structure with a lipophilic aglycone and a hydrophilic sugar side chain. A main characteristic of saponins is their foaming, soap-like behavior in aqueous solution, leading to their name (Latin *sapo* = soap) [3]. The aglycone moiety of saponins is generally referred to as the sapogenin, to which one or more sugar molecules are attached. Two classes of saponins are distinguished based on their type of aglycone: steroid (or steroidal) saponins and triterpene (or triterpenoid) saponins. Steroidal saponins consist of a steroidal aglycone, a C_27_ spirostane skeleton, generally comprised of a six-ring structure. Triterpenoid saponins consist of a triterpenoid aglycone, with a C_30_ skeleton and a pentacyclic structure.

Saponins exhibit a number of biological and pharmacological activities, such as anti-inflammatory, antifungal, antibacterial, cholesterol-lowering, anticancer, and adjuvant effects [4,5]. Moreover, due to their amphiphilic structure, many saponins have strong surface activity and have been used to increase cell membrane permeabilization and allow various (often polar) molecules to reach cytoplasm or nuclei [6].

Digitonin, from the foxglove plant, *Digitalis purpurea* (Plantaginaceae) is a steroidal saponin with a strong lytic activity on various biological membranes and cytotoxic activity against several cancer cell lines [7,8]. The activity of digitonin apparently depends on the presence of cholesterol in the membrane [9,10,11]. At particular low concentrations, digitonin can enhance the uptake of other compounds and therefore, can increase the toxicity of certain substances such as anticancer drugs [12], peptides [13], and natural secondary metabolites [14]. Previous studies have suggested that digitonin causes vesiculation and pore formation in cell membranes, resulting in the leakage of ions, small molecules, and proteins. We recently reported that digitonin penetrates into the inner layer of the membrane, then binding to cholesterol, so that both move to outer membrane layer; this eventually leads to membrane leakage [15]. However, the exact molecular mode of action of digitonin in causing cell membrane permeabilization is still unknown.

The aim of this work was to study the molecular interactions of digitonin with natural and artificial membranes present in red blood cells (RBCs) and in different lipid membrane models (large unilamellar vesicles/liposomes, LUVs, and giant unilamellar vesicles, GUVs). LUVs are vesicles composed of one or more lipid components (phospholipids, cholesterol). GUVs are large-sized vesicles, which are readily examinable by confocal microscopy and are employed for the same purpose of studying membrane permeability. The results clearly show that the membrane activity of digitonin depends on the presence and concentration of cholesterol. Formation of digitonin-cholesterol complexes is rapidly followed by membrane leakage or rupture as visualized by GUVs. Permanent pores are formed which allow solutions from outside the GUVs to pass to the inside. This work can explain why digitonin can be applied as a drug toxicity enhancer in a combination therapy as it facilitates the uptake of polar drugs. 

## 2. Results and Discussion

### 2.1. Digitonin Ruptures Red Blood Cells (RBCs)

**Figure 1:**
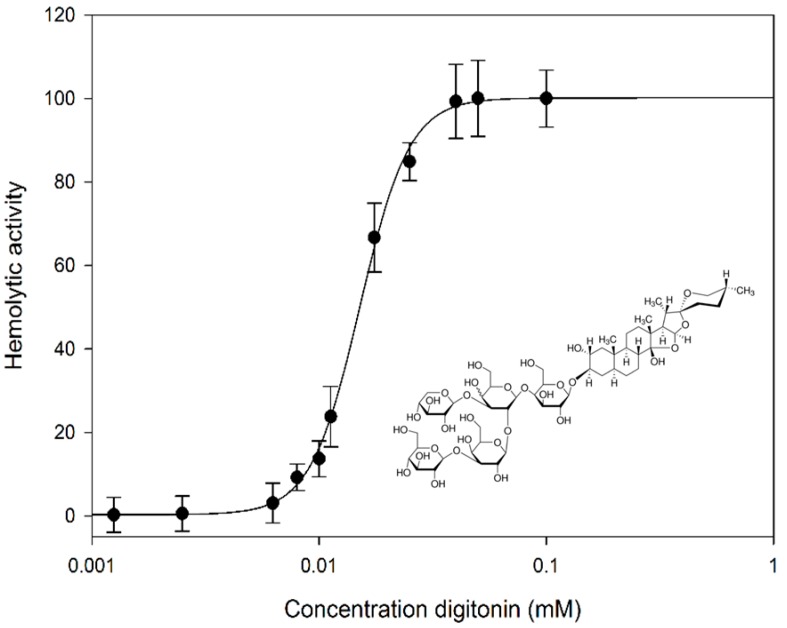
Dose-dependent hemolytic effect of digitonin on sheep RBCs. Digitonin concentrations were in the range from 0.001 to 0.1 mM. Hemolysis: IC_50_ 0.0151 mM. Data are expressed as means ± SD of hemolytic activity for three independent experiments. *Insert*: digitonin, structural formula.

Membrane lysis of natural cell membranes caused by digitonin was examined with RBCs. Defebrinated sheep red blood cells were mixed with differing concentrations of digitonin and the effect on membrane permeability was determined photometrically by measuring hemoglobin leakage. Salt solution (0.9%) and water were used as negative and positive controls. Digitonin affected sheep RBC membranes and ruptured them at an IC_50_ concentration of 0.0151 mM (Figure 1).

Red blood cells (RBC) are a suitable natural model to evaluate the effect of saponins on biological membranes. Saponins disrupt RBCs and this hemolytic activity depends on the chemical structure of the applied saponin (aglycones and sugar moieties). Many studies have shown that steroid saponins have stronger hemolytic effects than triterpenoid saponins [16]. Both aglycone types are strongly hemolytic if they carry only one sugar chain attached at C-3 (monodesmosides) rather than two sugar chains (bidesmosides) [17]. Our results support this finding: the monodesmosidic digitonin shows 30-times higher hemolytic activity than bidesmosides [12].

### 2.2. Digitonin Causes Calcein Leakage

**Figure 2:**
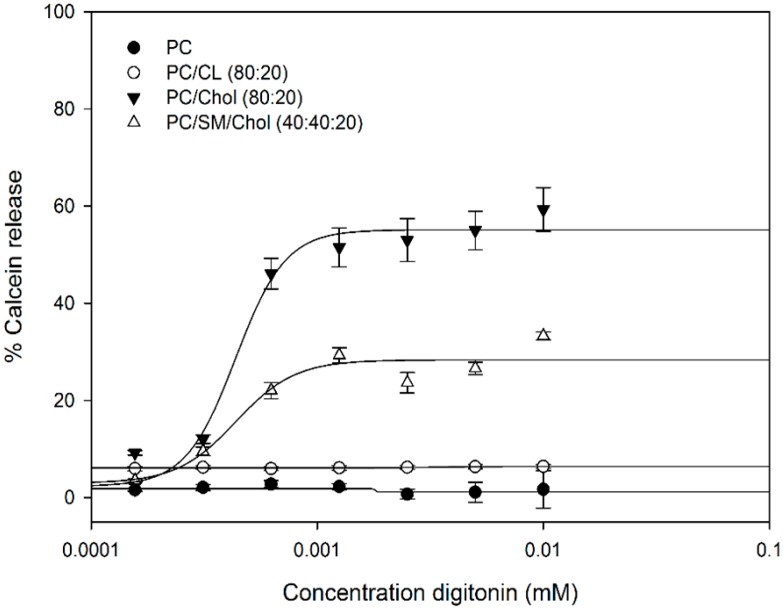
Calcein release from different LUVs induced by digitonin. Data are expressed as means ± SD of calcein release after 60 min for three independent experiments. PC: phosphatidylcholine; CL: cardiolipin; Chol: cholesterol; SM: sphingomyelin.

The specific interactions of digitonin with phospholipids and cholesterol were studied on artificial lipid vesicles loaded with the fluorescent calcein. Calcein leakage experiments were performed to quantify the extent of membrane permeabilization caused by digitonin (Figure 2). Several large unilamellar vesicles (LUVs) were prepared all differing in the composition of the membrane lipids phosphatidylcholine (PC), sphingomyelin (SM), cardiolipin (CL) (present in mitochondrial membranes), and cholesterol (Chol) in four different ratios: 100% PC, PC/Chol (80/20), PC/SM/Chol (40/40/20), and PC/CL (80/20). LUVs were incubated with different concentrations of digitonin and the release of calcein into the extravesicular solution was determined by fluorescence spectroscopy.

**Figure 3:**
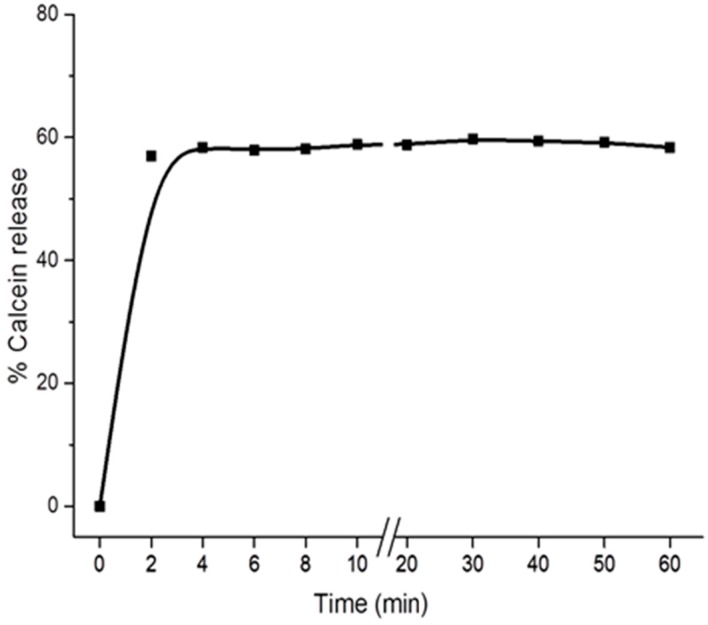
Kinetics of calcein release of PC/Chol (80:20) LUVs induced by digitonin. Intensity of fluorescence of calcein released from LUVs by 0.01 mM digitonin was measured continuously every 120 s for 1 h. The *inset* shows the percentage of calcein release in the range time of 0–10 min.

The results indicate that digitonin specifically interacts with cholesterol in membranes to induce vesicle leakage and releasing entrapped calcein to the solution. The intensity of extravesicular calcein fluorescence in the 100% PC and PC/CL (80/20) cases did not increase after incubation with digitonin for 24 h (data not shown). Digitonin immediately interacts with cholesterol-containing membranes inducing membrane leakage within less than 2 min, then remaining stable for more than one hour, as indicated by the steady level of extravesicular calcein fluorescence (Figure 3).

Cholesterol plays a crucial role for the activity of saponins on cell membranes. Saponins form complexes with cholesterol, leading to membrane leakage with a change in membrane permeability or pore formation allowing a free diffusion to and from the cytoplasm [11,18,19,20,21,22,23,24]. We used unilamellar vesicles as a membrane model to further elucidate the mechanism of this effect. We confirm that cholesterol is the target for monodesmosidic saponins in the membrane. Saponins appear not to interact with other lipid membrane components such as to phosphatidylcholine, sphingomyelin, or the mitochondrial cardiolipin. Both steroid and triterpenoid saponin induced a permeability change of LUV membranes containing cholesterol [9,18]. The sugar chain seems to play a crucial role in the hemolytic activity, the monodesmoside being more active in the membrane than bidesmosides [17]. The rate of membrane permeability induced by the saponins was relatively fast depending on saponin and cholesterol concentrations in the membrane.

### 2.3. Calcein Release Depends on Membrane Cholesterol Concentration

**Figure 4:**
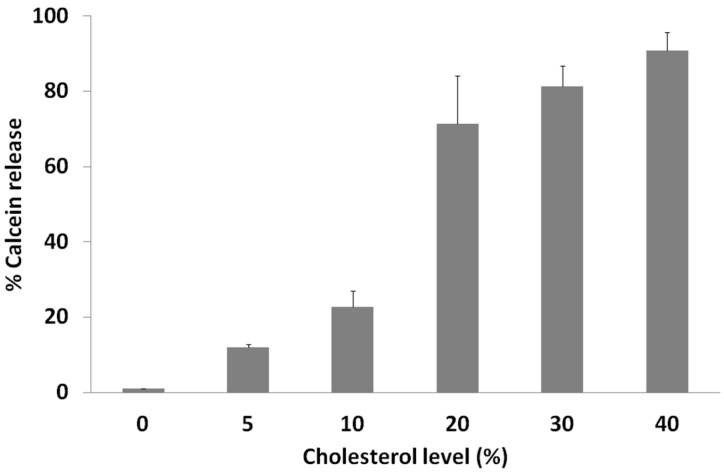
Influence of cholesterol concentration in artificial membranes on the lytic activity of digitonin. PC vesicles with different concentrations of cholesterol were incubated with 0.1 mM digitonin for 1 h. Data are expressed as means ± SD of percentage of calcein release for three independent experiments.

As shown before, cholesterol plays a crucial role for digitonin activity. We determined the influence of cholesterol concentration on membrane leakage (indicated by the intensity of calcein release) caused by digitonin (Figure 4).

One of the main functions of cholesterol in lipid bilayers and biological membranes is in increasing the packaging and rigidity of the membrane, maintaining the membrane lipids in the *liquid-ordered state*, causing the membrane to be laterally more condensed with increased packing density of the phospholipids. This increases the mechanical strength and decreases the permeability of the membranes [25,26]. Saponins readily change the structure of membranes that contain a high amount of cholesterol, as, e.g., RBCs with 45 mol % cholesterol. Hemolysis and calcein release data suggest the ratio of saponin: cholesterol complexation as being 1:1 [27,28,29]. This may explain the observed differences of saponin action against different cancer cell lines whose cholesterol content may differ.

### 2.4. Digitonin Affects Vesicle Size Only in the Presence of Cholesterol

**Figure 5:**
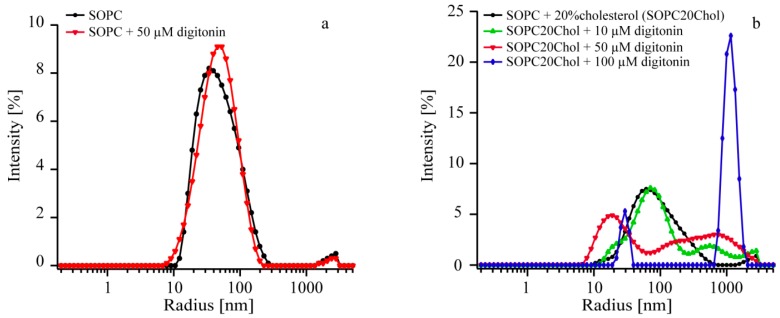
DLS profile of SOPC vesicle sizes with and without cholesterol after incubating with different concentrations of digitonin. (**a**) Control experiment: SOPC vesicles (black); SOPC + 50 µM digitonin (red); (**b**) SOPC20% cholesterol vesicles (SOPC20Chol) (black); SOPC20Chol + 10 µM digitonin (green); SOPC20Chol + 50 µM digitonin (red); SOPC20Chol + 100 µM digitonin (blue).

We applied dynamic light scattering (DLS) to elucidate possible interactions and effects of digitonin on vesicle membranes (with and without cholesterol) in terms of shape changes of vesicles. 1-Stearoyl-2-oleoyl-*sn*-glycero-3-phosphocholine (SOPC) vesicles without and with 20% cholesterol were prepared and incubated with different concentrations of digitonin for 30 min, then measured by DLS. Size distributions of SOPC-vesicles incubated with various concentrations of saponin are presented in Figure 5a and Table 1 (without cholesterol) and Figure 5b and Table 2 (with cholesterol).

**Table 1:** Asymmetric Gaussian fitting data of the vesicle size distribution plot for SOPC before and after digitonin incubation for 30 min. Corresponding curves are displayed in Figure 5a.

Sample	Before Incubation		Digitonin Concentration (µM)	After Incubation	
	r_max_ (nm)	FWHM (nm)		r_max_ (nm)	FWHM (nm)
SOPC	80	90	0	77	83
SOPC + 50	32	78	50	46	74

FWHM: full width at half maximum (FWHM).

**Table 2:** Asymmetric Gaussian fitting data of the vesicle size distribution plot for SOPC-Cholesterol (20 mol %) before and after digitonin incubation for 30 min. Corresponding curves are displayed in Figure 5b.

Sample	Before Incubation		Digitonin Concentration (µM)	After Incubation			
	Peak 1			Peak 1		Peak 2	
	r_max_ (nm)	FWHM (nm)		r_max_ (nm)	FWHM (nm)	r_max_ (nm)	FWHM (nm)
SOPC20Chol	77	93	0	63	65	ND	ND
SOPC20Chol + 10	58	154	10	71	99	470	1566
SOPC20Chol + 50	58	34	50	17	23	39	3038
SOPC20Chol + 100	51	252	100	30	10	1116	588

FWHM: full width at half maximum (FWHM); ND: not detected.

Shape and size of SOPC vesicles without presence of cholesterol are similar for both digitonin-treated and untreated vesicles, indicating that digitonin alone did not intercalate the vesicle membranes (Table 1 and Figure 5a). In the presence of cholesterol, shape and size of vesicles changed with increasing concentrations of digitonin, and vesicle size dramatically changed when incubating with the respective amount of digitonin, 0, 10, 50, and 100 µM. The comparison of data before and after incubation with digitonin implies that saponin has an influence on the size distribution when a concentration of at least 10 µM is applied (see Figure 5b and Table 2).

The data can be interpreted that at concentration less than 50 µM digitonin creates additional vesicles ranging from 200 to 1000 nm in size leaving many vesicles in the original size. However, from concentration of digitonin 50 µM upwards the number of vesicle ranging around 80 nm decreases and bigger vesicles are formed. With concentration 100 µM digitonin most vesicles have a size between 700 and 2000 nm and just a small peak at 29 nm is left. To account for the measurement errors, the intensity *vs.* radius data collected from the vesicle solutions without incubation were compared. When the same batch of vesicles is used the variations in the curve progression should be minimal. However, the maxima of the size distribution vary from 60 to 81 nm for the vesicle containing 20% cholesterol and 74 to 85 nm for SOPC vesicles. Measurements from different batches partly show shifted distributions or slightly different curve progressions. It can be seen that, the distributions show differences at a radius of 10 to 25 nm and between 2000 and 3000 nm. The later peak most likely derives from contaminations.

Nevertheless, DLS data definitely show that there is interaction between cholesterol containing vesicles and digitonin. Digitonin at a concentration 10 µM already induce membrane leakage and vesicles disruption and the effect increased with increasing of digitonin concentration [9,10,11]. The digitonin-cholesterol complexes cause morphological changes of the membrane forming an additional layer on the outside of the membrane causing membrane phase separation [15]. This leads to alteration of membrane structure and aggregate formation in various shapes and forms [30,31]. It was not possible to identify modes of interaction between vesicles and saponins only with this method, nor if permeabilization of membranes occurs at concentrations under 10 µM digitonin.

This provides an explanation for the synergistic activity of digitonin at low concentrations by facilitating the uptake of other drugs resulting in increased toxicity. For example, administration of 5 µM digitonin has been shown to increase the toxicity of monoterpenes, terpenes, polyphenols, and alkaloids [8,14] and the anticancer drug cisplatin [12] in various cancer cell lines. Administration of 20 µM digitonin increased the efficacy of cisplatin by 20% in clinically isolated lung perfusions [32]. However, administering precise concentrations of digitonin will be essential in approaching an efficient combination therapy with cytotoxic drugs.

### 2.5. Digitonin Affects GUV Membrane Permeability and Integrity in the Presence of Cholesterol

Fluorescence microscopy was employed to further investigate the interaction and permeabilization effect of digitonin on giant unilamellar vesicles (GUV) with a diameter of up to 100 µm. Dil stain was used to mark GUV membranes and Alexa Flour 488 fluorescence for coloring the extravesicular solution of GUVs. Membrane permeabilization was visualized by monitoring the presence of the dye inside of GUVs (black spheres). The kinetics and degree of membrane permeabilization can be quantified for individual vesicles.

**Figure 6:**
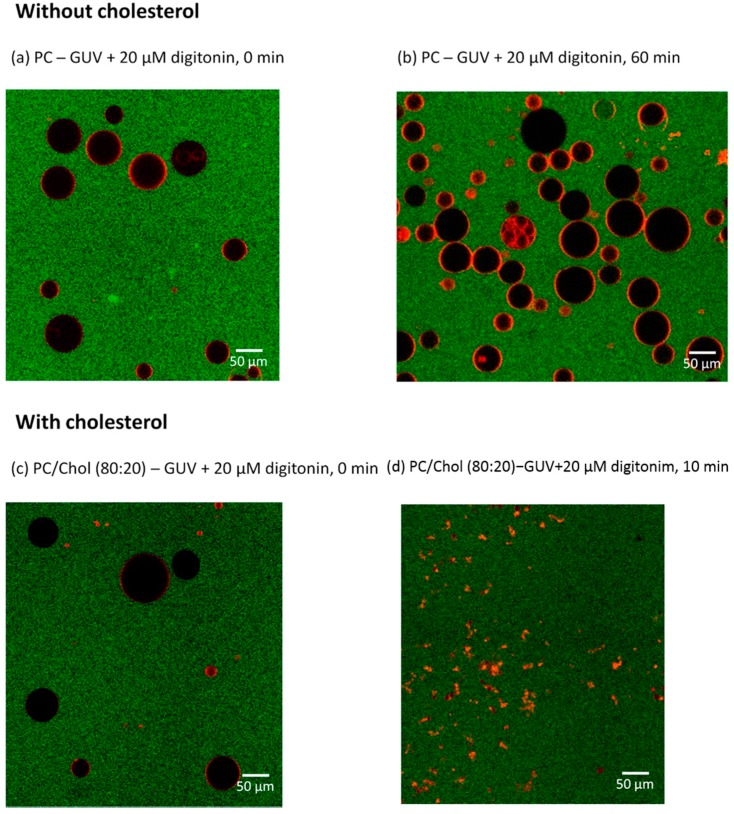
GUV membrane permeabilization by digitonin. (**a**) PC-GUVs shortly after applying 20 µM digitonin; (**b**) PC-GUVs after incubating with 20 µM digitonin for 60 min; (**c**) PC/Chol (80:20) GUVs shortly after applying 20 µM digitonin; (**d**) PC/Chol (80:20) GUVs after incubating with 20 µM digitonin for 10 min. Solution bathing GUVs (green), interior of GUVs (black), GUV membranes (red).

PC-GUVs were prepared either with or without cholesterol. The membrane permeabilization effect of digitonin was monitored for 60 min. Figure 6a–d show the effects of digitonin on GUV membranes, without and with cholesterol; (a) without cholesterol, shortly after adding the drug and (b) same, after 1 h of incubation; here, digitonin apparently cannot permeabilize the GUV membranes as these are still intact and there is no sign of influx through the membrane into the GUVs (*black interior*); (c) However, in the presence of cholesterol, digitonin affects membranes immediately; (d) after 10 min all GUVs were filled with green solution and most membranes were disrupted.

**Figure 7:**
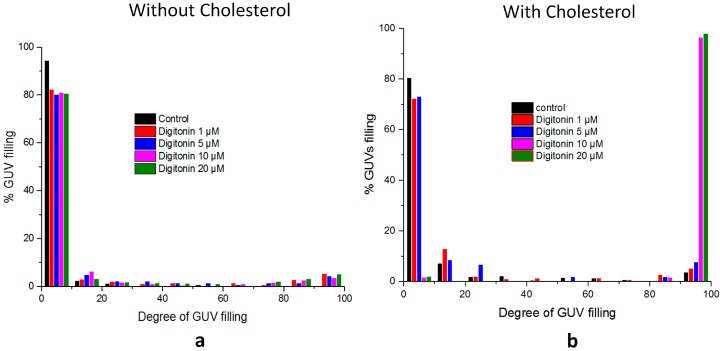
GUV filling upon incubating GUVs with different concentrations of digitonin. (**a**) PC-GUV with digitonin incubation 60 min; (**b**) PC/Chol (80:20)-GUV with digitonin incubation 10 min.

Membrane permeabilization and kinetics can be calculated by comparing the fluorescence intensity inside the individual GUVs with the background in the chosen incubation time. The degree of GUV filling represents the specific interaction between digitonin and the lipid membrane and also can be used to reveal the mechanism of membrane permeabilization. Different concentrations of digitonin were applied to determine their ability to induce membrane permeabilization with and without incorporated cholesterol. The results again show that digitonin can induce membrane rupture only in the presence of cholesterol in an all-or-none mechanism and that the concentration greatly affects the ability of digitonin to cause membrane disruption (Figure 7). 

### 2.6. Visualizing the Disrupting Effect of Digitonin on Individual PC/Chol (80:20) GUVs

**Figure 8:**
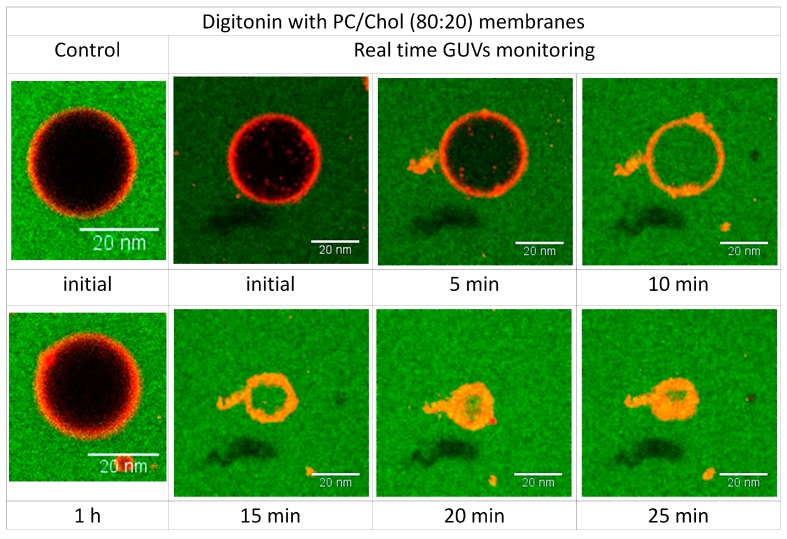
Time dependence of response of single PC/Chol (80:20)-GUV to 20 µM digitonin. Left images show GUV without digitonin as a control during the incubation time. The other images show time lapse interaction of 20 µM digitonin with PC/Chol (80:20)-GUV. Scale bars 20 nm.

The permeabilization kinetics was quantified by monitoring the permeability of individual GUVs in real time. The images were captured every 20 s for 1 h. The filling rate of PC/Chol-GUVs incubated with digitonin was very fast and most of them were filled after 10 min of incubation. The interaction of digitonin and cholesterol not only caused permeabilization but also led to vesicle rupturing (Figure 8; Videos 1 and 2 in Supplementary Materials).

**Video S1.** Time dependence of response of single PC-GUV to 20 µM digitonin. During the incubation time the GUV is not permeabilized by digitonin.

**Video S2.** Time dependence of response of single PC/Chol (80:20)-GUV to 20 µM digitonin. Digitonin leads to an increase of permeabilization (interior turns green, indicating extravesicular solution entering the GUV), and the vesicle eventually collapses resulting from membrane structural disintegration.

Giant unilamellar vesicles (GUV) are suitable for microscopic real-time monitoring of digitonin-cholesterol interactions [33,34,35,36]. We observed the response of single GUVs when exposed to various concentrations of digitonin. The results support the data obtained from hemolytic and calcein leakage experiments. Cholesterol is clearly shown as the target for digitonin and the effect of inducing membrane permeability in GUVs depends on the concentration of digitonin.

Digitonin acted by an all-or-none mechanism: in the absence of cholesterol no observable morphological changes of vesicles were seen even at a high concentration of ≥20 µM digitonin and even after more than 60 min. In contrast, inclusion of cholesterol in membranes leads to complex formation with digitonin leading to permanent pores and subsequent complete filling of vesicles. Strong permeability was evidenced at concentrations above 10 µM digitonin, when all vesicles had filled with green solution. The pore size as induced by the digitonin-cholesterol complex appears to be bigger than 2 nm based on the free diffusion of Alexa 488 dye having a diameter of 1.4 nm [37,38,39]. This explains the selectivity of substance passage through the cholesterol membrane by the action of digitonin.

Digitonin leads to increased vesicle size, and at higher concentrations to membrane rupture. GUV rupturing was monitored in real time. Interestingly, vesicles start to rupture after filling is complete, the vesicles then deflate as the solution seeps out from the vesicles. The pores formed by the digitonin-cholesterol complex seem to be retained for a longer period of time but with diameters of much more than 2 nm, leading to structural membrane instability. The membranes start to reorganize to become stable again forming small compact vesicles at the end. Increased levels of cholesterol in the membranes of above 20% will cause the vesicles to suddenly burst [40]. This result may explain the limitations of using digitonin as a toxicity enhancer: membranes are totally destroyed at higher concentrations of digitonin and cholesterol.

## 3. Experimental Section 

### 3.1. Materials

The following chemicals were employed: acetone (Zentralbereich Neuenheimer Feld, Heidelberg, Germany), Alexa Flour 488 (LifeTechnology/Thermo Fisher, Darmstadt, Germany), ammonium hydroxide (Sigma Aldrich, Steinheim, Germany), bovine serum albumin (BSA) (Sigma Aldrich), cardiolipin (heart, bovine) (CL) (Avanti Polar Lipids, Inc., Alabaster, AL, USA), chloroform (Sigma Aldrich), cholesterol (Avanti Polar Lipids, Inc.), desalting column PD-10 (GE Healthcare, Garching, Germany), digitonin (Sigma Aldrich), Dil stain (dioctadecyltetramethyl-indocarbocyanine perchlorate) (LifeTechnology/Thermo Fisher), 1,2-dipalmitoyl-*sn*-glycero-3-phosphocholine (DPPC) (Avanti Polar Lipids, Inc.), Ethanol (Zentralbereich Neuenheimer Feld), methanol (Zentralbereich Neuenheimer Feld), Milli-Q H_2_O (Thermo Electron LED GmbH, Niederelbert, Germany), L-α-phosphatidylcholine (egg, chicken) (PC) (Avanti Polar Lipids, Inc.), Phospholipids B kit (Wako Chemicals, Neuss, Germany), Sphingomyelin (egg, chicken) (Avanti Polar Lipids, Inc.), 1-stearoyl-2-oleoyl-*sn*-glycero-3-phosphocholine (SOPC) (Avanti Polar Lipids, Inc.), Texas Red-DHPE (Avanti Polar Lipids, Inc.), Triton X-100 (Sigma Aldrich).

### 3.2. Hemolytic Activity

Defribrinated sheep blood was centrifuged at 3000 rpm for 5 min and red blood cells were then resuspended in isotonic NaCl solution to obtain a 1% erythrocyte solution. Serial dilutions of digitonin were prepared in an isotonic NaCl solution. 500 µL of sample was mixed with 500 µL of erythrocytes in Eppendorf tubes and then incubated for 1 h at 37 °C. After incubation the tubes were centrifuged at 1000 rpm for 5 min and then the supernatant was transferred into 96-well plate. The absorbance of hemoglobin in the supernatant was measured at 544 nm with a Biochrom UVM-340 microplate reader (Biochrom, Cambridge, UK). As a positive control 5% Triton X-100 was employed and an isotonic NaCl solution as a negative control. Measurements were replicated three times for each digitonin concentration. Hemolytic activity was calculated with the following formula [8].

(1) $$\%\;\mathrm{hemolysis}\;=\;\frac{\mathrm{absorbance}\;\mathrm{of}\;\mathrm{sample}\;–\;\mathrm{absorbance}\;\mathrm{of}\;\mathrm{blank}}{\mathrm{absorbance}\;\mathrm{of}\;\mathrm{positive}\;\mathrm{control}}\;\times \;100$$

### 3.3. Preparation of Lipid Vesicles and Entrapment of Calcein

Large unilamellar vesicles (LUVs) were prepared by hydration of a thin lipid film, according to the Bangham method. The desired membrane lipids, in appropriate amounts, were dissolved in chloroform, mixed properly in a round-bottom flask, and then dried under a stream of N_2_ gas. The solvent was completely removed by keeping the sample in the vacuum desiccator connected to a rotary vacuum pump for more than 12 h. To prepare LUVs containing calcein, lipids were resuspended to a concentration of 4 mg/mL in 1 mL solution containing 80 mM calcein in water (pH 7.4, adjusted with NaOH) continuing with vortexing for 30 s several times at room temperature. Next, the lipid-calcein suspension was subjected to six cycles of freezing and thawing in liquid N_2_. Afterwards the solution was extruded 31 times through double-stacked polycarbonate membrane (pore size, 100 nm) using a two-syringe extruder in a LiposoFast liposome extruder (Avestin, Ottawa, ON, Canada) until the solution became transparent. The untrapped calcein was removed from LUVs solution through a PD-10 desalting column (GE Healthcare Life sciences, Piscataway, NJ, USA) equilibrated with outside buffer. The lipid concentration was estimated with the Phospholipids B kit (Wako Chemicals, Richmond, VA, USA). Fluorescence intensities of calcein entrapment in LUVs were measured at room temperature at excitation wavelength 490 nm and emission wavelength 520 nm [41].

### 3.4. Permeabilization of LUVs (Calcein Release Assay)

Permeability activity of digitonin at the LUVs was determined by measuring the intensity of calcein fluorescence released into solution from LUVs after introduction of digitonin. The black 96-well plate was blocked with 10% bovine serum albumin (BSA) for 1 h at room temperature to avoid nonspecific interaction between plate material with digitonin and vesicles. The BSA solution was removed from the plate, continuing with washing several times with sterile water, then dried. Following this, 100 µL of LUVs with entrapped calcein was added to each well. Different concentrations of digitonin were applied immediately before measurements with a Tecan Infinite M200 plate reader (Tecan, Männendorf, Switzerland) at fluorescence emission wavelength of 520 nm and excitation at 495 nm. The increased fluorescence intensity of calcein was monitored with time until a stationary state was reached. As a positive control 5% Triton X-100 was employed and buffer as a negative control. The percentage of calcein release from vesicles induced by digitonin was calculated from:

(2) $$\%R\;=\;100\frac{F_{f}\;–F_{i}}{F_{m}\;–\;F_{j}}$$

where *F_f_* is the calcein fluorescence intensity at a specific time after incubation with digitonin, *F_i_* the initial calcein intensity before adding digitonin, and *F_m_* the maximum intensity of calcein upon adding 5% Triton X-100 [41].

### 3.5. Preparation of Giant Unilamellar Vesicles (GUVs)

Giant unilamellar vesicles (GUVs) were prepared by the electroformation method. The desired lipid composition with and without cholesterol was mixed with Dil stain to visualize GUV membrane rims under the fluorescence microscope. The lipid mixture at 1 mg/mL in chloroform was spread onto two platinum wire electrodes at 5 µL per each wire and the solvent was evaporated for one minute. After the solvent had completely evaporated, the platinum wires were immersed in a chamber containing 300 mM sucrose solution and then the unit was connected to a power generator. The electroformation proceeded at 2.3 V and 10 Hz for 2 h, then followed by 30 min at 2 Hz at room temperature to release the GUVs from the electrode wires. The 40 µl of GUVs electroformed in 300 mM sucrose were transferred to 400 µL PBS onto an eight-chamber slide (Lab-Tek™ II Chamber Slide™ System, Nunc™, Waltham, MA, USA) that was previously blocked with 2 mg/mL bovine serum albumin [42].

### 3.6. Membrane Permeability Assays

For GUVs permeabilization measurements, Alexa Flour 488 was added and mixed properly with PBS containing digitonin solution before GUVs were added into it. The Alexa Flour 488 fluorescence can differentiate between solution from the outside and inside of GUVs. The degree of permeabilization (filling kinetics) was determined by collecting pictures of several GUVs in different sample conditions every 20 s for 1 h. GUV images from several regions were taken after 2 h incubation with or without digitonin to reach the final extent of vesicle permeabilization. Permeabilized and nonpermeabilized GUVs were counted and analyzed with homemade GUV detector software and ImageJ [42,43,44].

### 3.7. Confocal Microscopy (CM) and Fluorescence Correlation Spectroscopy (FCS)

Membrane permeabilization by digitonin was monitored with LSM710 microscope with a C-Apochromat 40×/1.2W Corr M27 water immersion objective (Zeiss, Oberkochen, Germany) in multitrack modus. The green channel consisted of excitation light from an Ar-ion 488 nm and a 505–530 nm band-pass filter. The red channel consisted of excitation light from a He-Ne 561 nm with 633 nm excitation laser and a 650 nm long-pass filter. Fluorescence cross-correlation spectroscopy (FCCS) measurements were performed at 22 °C using a Confocor 3 module. Photon arrival times were recorded with a hardware correlator Flex 02-01D/C. We repeatedly scanned the detection volume with two perpendicular lines through the equator of a GUV (the distance between the two lines, *d*, was measured by photobleaching on a film of dried fluorophores). The data was analyzed using homemade software [42]. We binned the photon stream in 2 μs and arranged it as a matrix such that every row corresponded to one line scan. We corrected for membrane movements by calculating the maximum of a running average over several hundred line scans and shifting it to the same column. We fitted average overall rows with a Gaussian and we added only the elements of each row between −2.5 s and +2.5 s to construct the intensity trace. We computed the autocorrelation and spectral and spatial cross-correlation curves from the intensity traces and excluded irregular curves resulting from instability and distortion. The auto- and cross-correlation functions were then fitted with a nonlinear least-squares global fitting algorithm, as described by García-Sáez *et al*. [42] and Bleicken *et al*. [37].

### 3.8. GUV Image Analysis

Permeability of GUVs is defined by influx of colored solution through the GUV membranes. The percentage of GUV filling was calculated according to the equation below. The threshold for nonpermeabilized GUVs was set to <15%. Several hundred GUVs were analyzed per experiment.

(3) $$\left[\frac{\left(F_{t}^{in}\;–\;F_{0}\right)}{\left(F_{t}^{out}\;–\;F_{0}\right)}\right]\;\times \;100$$

in which $F_{t}^{in}$ is the average fluorescence intensities inside a GUV, $F_{t}^{out}$ the average fluorescence intensities outside a GUV, and *F*_0_ the background fluorescence at time *t*. 

To quantify digitonin binding to GUV membranes, the fluorescence intensity at the vesicle rim (*F*_rim_) was calculated with ImageJ using the plug-in radial profile plot. Intensity was plotted as *F*_rim_/*F*_back_, where *F*_back_ is the background intensity outside the GUVs.

In the kinetics experiments, images were recorded every 20 s and changes in the fluorescence intensity inside GUVs were analyzed over time as

(4) $$F_{t}^{N}\;=\frac{\left(F_{t}^{in}\;–\;F_{0}\right)}{\left(F_{t}^{out}\;–\;F_{0}\right)}$$

$F_{t}^{N}$ is the normalized fluorescence intensity at time *t*.

To calculate the initial *A*_0_ and relaxed *A*_relax_ permeabilized area of individual GUVs, as well as the relaxation time τ*_relax_*, we used a multiexponential fitting described by:

(5) $${F(t)}_{in}^{N}\;=\;1\;–\frac{–t}{e^{\text{τ}\mathrm{flux(t)}}}$$

where the influx rate, τ*_flux_*, decreases with time (the initial pore size relaxes to a smaller structure) according to

(6) $${\text{τ}}_{\mathrm{flux}}(t)\;=\;\frac{\text{V}}{A(t)\;\times \;\frac{D}{m}}$$

where *V* is the vesicle volume, *D* the dye diffusion coefficient, *m* the membrane thickness, and *A* the total permeabilized area, which varies with time according to

(7) $$A(t)\;=\;A_{\mathrm{relax}}\;+\;(A_{0}\;–\;A_{\mathrm{relax}})\;\times \;\frac{–t}{e^{\text{τ}\mathrm{relax}}}$$

Membrane thickness was assumed to be 4.5 nm. The diffusion coefficient of cyt *c*-al488 was 196 μm^2^/s (196 ± 27 μm^2^/s) as calculated by fluorescence correlation spectroscopy (FCS) [37].

### 3.9. Size Measurement by Dynamic Light Scattering (DLS)

In order to get an overall idea about the effects of digitonin on the phospholipid bilayer membrane, the size change of small unilamellar vesicles was measured using a dynamic light scattering (DLS) technique. Different concentrations of digitonin were added to the suspension of vesicles with or without cholesterol incorporation and the size distribution was measured at room temperature before and after an incubation of 30 min. The 900 µL of vesicle solution (0.5 mg/mL) was inserted in a disposable cuvette (Roth, Karlsruhe) and measured with 11 scans of 10 s in a DLS Zetasizer Nano ZS (Malvern Instruments, Malvern, UK) at 21 °C. Afterwards 100 µL of digitonin was added and after incubation for 30 min at room temperature, the solution was measured once more. For the control sample instead of digitonin, 100 µL of filtered Milli-Q water was added to the vesicle solution and further processed as described above. The data was analyzed using the asymmetric Gaussian function (ExpModGauss) multipeak fitting 2 package for IGOR PRO software (Wavemetrics, OR, USA).

## 4. Conclusions 

In the present study we attempt to understand the molecular mechanism of digitonin on membranes in regard to its ability to change membrane permeability. The results support the essential role of cholesterol in explaining the activity of digitonin on biological and artificial membranes [9,13]. Digitonin induces membrane permeability or causes membrane rupturing only in the presence of cholesterol in an all-or-none mechanism. This effect depends on the concentrations of both digitonin and cholesterol. At low concentrations, digitonin induces membrane permeability while keeping the membrane intact. Digitonin thus can act synergistically in combination with other drugs to enhance their toxicity because their uptake is facilitated. Administering precise concentrations of digitonin will be essential in approaching an appropriate combination therapy. This study extends and complements the numerous related studies on the activity and mode of action of digitonin on membranes [9,10,11,12,13,14,15].

## Supplementary Materials

Supplementary materials can be accessed at: http://www.mdpi.com/1420-3049/20/11/19682/s1.
